# A Pilot Study on the Relationship between Cardiovascular Health, Musculoskeletal Health, Physical Fitness and Occupational Performance in Firefighters

**DOI:** 10.3390/ejihpe12110120

**Published:** 2022-11-21

**Authors:** Jaron Ras, Denise L. Smith, Elpidoforos S. Soteriades, Andre P. Kengne, Lloyd Leach

**Affiliations:** 1 Department of Sport, Recreation and Exercise Science, Faculty of Community and Health Sciences, University of the Western Cape, Cape Town 7535, South Africa; 2 Health and Human Physiological Sciences, Skidmore College, Saratoga Springs, New York, NY 12866, USA; 3 Healthcare Management Program, School of Economics and Management, Open University of Cyprus, 2200 Nicosia, Cyprus; 4 Environmental and Occupational Medicine and Epidemiology (EOME), Department of Environmental Health, Harvard T.H. Chan School of Public Health, Harvard University, Boston, MA 02138, USA; 5 Non-Communicable Diseases Research Unit, South African Medical Research Council, Cape Town 7500, South Africa

**Keywords:** firefighters, CVD risk factors, musculoskeletal health, physical fitness, occupational performance

## Abstract

Firefighters’ face life threatening situations and are frequently exposed to numerous physical, chemical, biological, ergonomic and psychosocial hazards. The purpose of this pilot study was to investigate the feasibility of conducting a large-scale study on cardiovascular and musculoskeletal health, physical fitness and occupational performance of firefighters. We conducted a cross-sectional pilot study by recruiting 36 firefighters. A researcher-generated questionnaire and physical measures were used to collect data on sociodemographic characteristics, cardiovascular and musculoskeletal health, physical fitness and occupational performance using a physical ability test (PAT). We documented a high equipment and intra-assessor reliability (r > 0.9). The potential logistic and/or administrative obstacles in the context of a larger study were discerned. Data were successfully retrieved using available equipment and survey instruments. Hypertension (30.6%) dyslipidaemia (33.3%), obesity (36.1%) and physical inactivity (66.7%) were the most prevalent cardiovascular disease risk factors. A significant difference between genders in total PAT completion time was also seen (*p* < 0.001). Cardiorespiratory fitness, lean body mass, grip strength and leg strength were significantly associated with occupational performance (*p* < 0.001). The pilot study supports the larger study feasibility and verified equipment and assessors’ reliability for research. Cardiovascular health, musculoskeletal health and physical fitness may be related to PAT performance.

## 1. Introduction

Firefighting is a hazardous occupation where firefighters routinely face life threatening situations, and are frequently exposed to high temperatures, toxic chemicals and fumes and many other hazards [1,2]. This necessitates that firefighters wear heavy insulated personal protective equipment (PPE), which places additional physical strain on firefighters [3,4]. In addition, firefighters have been shown to have multiple comorbidities, predisposing them to sudden cardiac events [1,5,6,7]. Moreover, many firefighters have low musculoskeletal health, and unfavourable physical fitness which restricts their occupational performance while on duty [8,9]. 

Over 45% of firefighters’ on-duty fatalities are related to cardiovascular disease incidents [1]. Moreover, lower extremity, as well as lower back injuries, are highly prevalent in this population, negatively affecting their occupational performance [8,9]. To assist in maintaining good cardiovascular and musculoskeletal health, firefighters are expected to maintain a satisfactory level of physical fitness [10,11]. However, studies have shown that many firefighters’ physical fitness levels do not appear sufficient to cope with the strains of their profession, further predisposing an already at-risk population to early career morbidity, job disability and higher mortality [12,13,14]. 

Firefighters have been reported to have a high prevalence of cardiovascular disease (CVD) risk factors, with many firefighters having multiple comorbid CVD risk factors [15,16,17,18,19,20,21]. Furthermore, firefighters are reported to have a poor attitude toward health, with many firefighters opting to remain sedentary and engage in unhealthy dietary habits [22,23,24]. Several firefighters are physically inactive during their leisure times and frequently suffer from musculoskeletal injuries [25]. Given the reported high levels of physical inactivity and relatively high rates of musculoskeletal injuries, research and associated policies are needed to mitigate the risk of cardiac events and musculoskeletal injuries among firefighters while on duty. 

Therefore, the purpose of this pilot study was to investigate the feasibility of conducting a large-scale study on the cardiovascular health, musculoskeletal health, physical fitness and occupational performance among operational active firefighters. Specific aims were to explore possible logistic and/or administrative obstacles of a larger study; determine the intra-tester reliability of selected research equipment and instrument (questionnaire); determine the prevalence of CVD risk factors, musculoskeletal injuries, cardiorespiratory fitness and physical ability test (PAT) failures; and to explore the extent to which CVD risk factors and health metrics, musculoskeletal health and physical fitness are related to occupational performance in firefighters. 

## 2. Materials and Methods

This pilot study used a quantitative, non-experimental, cross-sectional and correlational research design. The pilot study took place between May and June 2022 at one of the largest fire stations in the Cape Town Metropolitan area. 

### 2.1. Participants 

The study included all full-time male and female firefighters between the ages of 18 and 65 years. Part-time, volunteer and firefighters on leave were excluded from this study. In addition, administrative staff, or firefighters on administrative duty, due to illness or injury, were excluded from the pilot study. Firefighters were approached at the fire station to participate in the study. Information on the study was provided to each firefighter, before informed consent was signed by each participant. 

### 2.2. Instrument and Tester Reliability and Validity

Prior to commencement of the pilot study, an initial trial was conducted where the assessors measured the reliability of their measurement techniques for all testing procedures used. In order to ensure intra-assessor reliability and validity, a minimum test–retest reliability coefficient of 0.8 was required prior to the commencement of the study to ensure tester reliability and was standardized across all measurements [26,27,28]. Each tester was tasked to perform one measurement for the duration of data collection to ensure test–retest reliability and data accuracy. Five successive measurements were obtained on all measurable study variables using standard and precision research equipment (CardioChek Plus analyzer, Omron Healthcare, Ltd., Hoofddorp, The Netherlands, blood pressure cuff and the Tanita© BC-1000 Plus BIA scale) and reliability coefficients were calculated. The technical error of measurement (TEM) was within acceptable parameters for the research being conducted [26,27,28]. The IPAQ was used to measure physical activity, which was shown to be a reliable and valid tool [29,30]. Systematic error of the equipment was tested and an accuracy rating of less than 5% was considered appropriate for research procedures. 

### 2.3. Research Procedures

A researcher-generated questionnaire was used to obtain information on sociodemographic data, cardiovascular health, musculoskeletal health, and lifestyle habits. The musculoskeletal health section of the questionnaire was based on the Cornell Musculoskeletal Discomfort Questionnaire [31]. Questions on physical activity were based on the International Physical Activity Questionnaire (IPAQ) [29]. 

### 2.4. Descriptive Measures

Stature was measured using a portable stadiometer (Seca model 700, Gmbh & Co., Hamburg, Germany), standing barefoot on a level floor with the heels together and the heels, buttocks and upper back touching the stadiometer rod. A Tanita© BC-1000 Plus bioelectrical impedance (BIA) analyzer was used to obtain body composition data, which included weight, lean body mass (LBM), fat mass, body fat percentage (BF%) and body mass. For the BIA assessment, firefighters were requested to wear minimal clothing, to stand upright, barefoot, and stationary on the scale. Waist circumference was measured at the point of the belly button [32]. Hip circumference was obtained at the level of the greatest posterior protuberance of the buttocks. Blood pressure was measured using the Omron Healthcare, Inc. M6 comfort intelligence automatic blood pressure monitor. Firefighters were asked to remain in a quiet seated position for 5 min prior to testing, with the left arm elevated onto the testing table. The midline of the bladder of the blood pressure cuff was placed over the brachial artery to ensure accurate and consistent readings. The participants blood pressures were obtained thrice, with at least two-minute intervals between measures. Total cholesterol and non-fasting blood glucose concentration was measured using a CardioChek^®^ Plus analyzer, which has been shown to be accurate and reliable within industry standards [33]. The test entailed a finger prick, wherein the initial blood droplet was wiped off, and a second drop of blood was used for testing purposes.

### 2.5. Classification of Cardiovascular Disease Risk Factors

Age, as a risk factor, was classified as males over the age of 45 years and females over the age of 55 years [32]. Obesity was classified as a body mass index above 30 kg·m^−2^, central obesity was classified as a waist circumference above 102 cm for men and above 88 cm for woman [32]. Hypertension was classified as either a SBP above 140 mm Hg or a DBP blood pressure above 90 mm Hg or both or confirmed by a physician [32]. Dyslipidaemia was classified as a total cholesterol concentration above 5.18 mmol·L or previously confirmed by a physician, and diabetes classified as a non-fasting blood glucose concentration above 11.1 mmol·L or previously confirmed by a physician. Cigarette smokers were classified as those that are current smokers or who have quit within the last six months [32]. Physical inactivity was classified as firefighters who exercised less than three days a week for at least 30 min [32]. A family history was classified as those that had a family history of myocardial infarction, coronary revascularization, or sudden cardiac death before 55 years in father or other first degree male relative, or before 65 years in mother or other first degree female relative [32].

### 2.6. Heart Rate Variability 

Heart rate variability was measured at rest using the Polar™ H10 heart rate monitor. The equipment was moistened with room temperature water and fitted to the center of the participant’s chest, directly in line with the xiphoid process of the sternum. The participant was asked to maintain a quiet seated position for five minutes before the measurement was taken. The participant’s HRV was then recorded over a five-minute period, following the five-minute rest period, giving a total test time of 10 min. The HRV data were analyzed using the Kubio© Software version 3.4.3. The standard deviation of all normal-to-normal (NN) intervals (SDNN), root-mean-square of successive differences (rMSSD), low-frequency, high frequency ranges and also the ratio (LF/HF) are the most widely used HRV indices, and were used as main outcome measures for this pilot study [33,34,35]. 

### 2.7. Physical Fitness

Data components of physical fitness were captured using a researcher-generated data collection sheet and the administration of physical tests and handgrip and back and leg dynamometer. 

#### 2.7.1. Cardiorespiratory Fitness

Cardiorespiratory fitness was estimated using the non-exercise method by applying the following formula: VO2max = 3.542+ (−0.014 × Age) + (0.015 × Body Mass [kg]) + (−0.011 × Resting Heart Rate) [36]. 

#### 2.7.2. Handgrip Strength

Handgrip strength was assessed with a Takei^®^ 5401-C handgrip dynamometer for upper body muscular strength and measured using a following standardized procedures from the American College of Sports Medicine (ACSM) [32]. Manufacturer accuracy for the handgrip ± 2.0-kg force (kgf). The grip bar was adjusted so the second phalangeal joint fit snugly under the handle. The dynamometer was set to zero. The firefighters were asked to hold the handgrip dynamometer in line with the forearm and the level of the thigh, and away from the body. The firefighters were asked to squeeze with as much force as possible, without holding their breath. The procedure was repeated twice and the highest reading of the two measures was recorded. 

#### 2.7.3. Leg Strength Dynamometer

Leg strength was measured with a Takei^®^ back and leg strength dynamometer. Manufacturer accuracy for the back and leg strength dynamometer ± 6.0 kgf, respectively. To assess leg strength, the firefighters were asked to remain upright on the base of the dynamometer with their feet shoulder width apart. Firefighters were requested to allow their arms to remain in an extended position with their hands grasping the dynamometer with their palms in prone position holding the bar. The chain was adjusted to ensure that each firefighters’ knees was in 110 degrees of flexion, which was approximately the midpoint of the patella tendon. The firefighters were instructed to pull as forcefully as possible on the chain, while attempting to straighten their knees. The procedure was repeated twice and the highest reading of the two was recorded.

#### 2.7.4. Push-Ups

For upper body muscular endurance, the push-ups test was used [32]. The procedures for the push-up test were conducted in accordance with the ACSM guidelines [32]. Males were requested to position themselves in the standard prone position, with their hands positioned forward and under the shoulder, back in a straight position, head level and their toes as the pivotal point. Females were requested to perform the modified push-up position, with their hands shoulder width apart, head up, back straight, with their legs together, lower leg in contact with the mat, ankles in plantar flexion and their knees acting as the pivotal point. Firefighters were required to raise the body off the mat fully extending their elbow joints and returning to the down position. A hedgehog was placed under the chest of firefighters to maintain consistency when counting each repetition. The test was stopped when firefighters could not perform an additional push-up, or when two consecutive push-ups were performed incorrectly.

#### 2.7.5. Sit-Ups

For abdominal muscular endurance, the sit-ups test was used [32]. The firefighters were asked to lay supine on the mat with their knees at 90 degrees flexion, with their hands across the shoulders, elbows pointing forward [37]. The firefighters were required to touch their knees with their elbows and then go back so the shoulders touch the floor. The number of repetitions performed were recorded. The test was ended when the firefighters experienced exhaustion, denoted as the inability to perform another repetition [37]. 

#### 2.7.6. Flexibility

Lower back and hamstring flexibility was assessed with the sit-and-reach method. The ACSM [32] guidelines for the sit-and-reach were used when conducting this test. The firefighters were asked to position themselves in a seated position, barefoot, where their knees were completely extended and the soles of the feet flat against the sit-and-reach box and the inner edges of the soles roughly 15.2 cm apart. Each firefighter was asked to inhale and, when exhaling, to drop the head between the arms and slowly reach as far forward as possible, holding the stretched position for approximately two seconds. Firefighters were given three attempts, and the most distant point reached with the fingertips was recorded.

#### 2.7.7. Shoulder Reach Flexibility Test

The shoulder reach flexibility test was used to assess shoulder flexibility. The test required firefighters to touch the fingertips of each hand behind the back [38]. The firefighters were asked to flex one shoulder, externally rotate the humerus and flex the elbow while keeping the hand in a prone position against the torso. For the opposite arm, the shoulder joint was extended fully, internally rotated the humerus and flexed the elbow joint with the hand placed in the prone position, facing away from the torso [38]. Flexibility was graded based on whether the fingertips were touching.

### 2.8. Occupational Performance 

The PAT was used to assess operational performance and was conducted according to the testing protocol of the City of Cape Town Fire and Rescue Service (CoCTFRS). The PAT is a test of simulated firefighting tasks and is graded on the time taken for each test (maximum time to complete task), and includes six simulation tasks, namely: (1) step-ups (≤90 s); (2) charged hose drag-and-pull (≤180 s); (3) ladder raise and extension (≤60 s); (4) equipment carry (≤60 s); (5) forcible entry (≤60 s); and (6) rescue drag (≤60 s). Firefighters are required to complete the simulation protocol in under 9 min (540 s) in order to pass, and are required to pass each task in under the recommended time, or the task will be deemed a failure. Firefighters are allowed 20 s recovery between tasks. 

#### 2.8.1. Step-Ups

The firefighters were required to place a high-rise pack onto their shoulders, which consisted of two 20 kg weights, made up of in a twin donut method. They were further required to perform 30 step-ups on a 200 mm platform. The step-up required firefighters to place both feet onto a platform for each repetition, and back onto the ground into the starting position. 

#### 2.8.2. Charged Hose Drag and Pull

This task required the firefighters to drag a charged 45 mm line 27 m and then pull the remainder of the charged line a further 15 m. To simulate a charged hose, a 45 mm line tied to a tyre was preferred. The event required that firefighters place the hose-line over their shoulder or across the chest and advance the hose to the 27 m mark. Thereafter, the firefighters dropped to at least one knee, or in a seated position and pulled the hose-line to the 15 m mark. 

#### 2.8.3. Ladder Raise and Extension

In this task, firefighters were asked to walk a ladder six meters toward the building, raise a 7–8 m aluminum ladder using every rung, in a hand-over-hand fashion, until stationary. Immediately thereafter, the firefighters walked to the second pre-position and, using the hauling line, hoisted a 35 kg drum, pulling down the line hand-over-hand, until the fly section reached the pulley and then lower the ladder once again. The firefighters then walked back the ladder and lowered the ladder using the hand-over-hand technique, returning the ladder to the original position. 

#### 2.8.4. Equipment Carry

The equipment carry involved the firefighters carrying two foam drums weighing 25 kg each for a distance of 50 m. Firstly, the firefighter removed two foam drums from a 1.2 m high platform, one at a time, and placed them on the ground. The firefighters then proceeded to carry the drums 25 m around the first marked position, around the cone, and back to the starting point, walking another 25 m. Upon returning, the firefighters placed the foam drums back onto the platform, one at a time.

#### 2.8.5. Forcible Entry

The forcible entry event required firefighters to pick up a 6 kg sledgehammer and strike the tyre to drive the tyre a distance of 600 mm. 

#### 2.8.6. Rescue Drag

This event required firefighters to grasp an 80 kg tyre on the shoulders of the harness and drag the tyre 11 m to a prepositioned mark, perform a 180-degree turn, around the mark, and continue an additional 11 m toward the finish line. 

### 2.9. Statistical Analysis

The data were analyzed using SPSS^®^ software, version 28 (Chicago, IL, USA). The data were collected, coded and cleaned for errors using the double entry method on Microsoft Excel. Descriptive statistical analyses, such as the mean, standard deviation, frequencies, and percentages were performed. To assess the test–retest reliability of the equipment and the inter- and intra-assessor reliability, Pearson’s correlation was used. A test for normality was performed using the Shapiro–Wilk test, and indicated the data were not normally distributed. Thereafter, inferential statistics, consisting of the Mann–Whitney-U test and Kruskal–Wallis-H for differences and the Spearman’s Correlation coefficient for continuous variables were performed. A *p*-value of <0.05 was used to indicate statistical significance.

## 3. Results

### 3.1. Recruitment of Participants

The questionnaire and the static physical measures took approximately 15 to 20 min each to complete, while the physical fitness tests took approximately 10 to 15 min to complete, and the PAT took approximately 5 to 15 min to complete. Overall, the average time for the completion of the testing battery was 40 to 55 min per firefighter. However, three to four firefighters could be tested concurrently and up to four firefighters were allowed to complete a testing battery in an hour, indicating that such data collection is feasible for the larger study. The pilot study provided the researchers with the opportunity to recruit participants to participate in the larger study and allowed researchers to plan and coordinate with staff to test all participants. The response rate was very good, as all the firefighters who were approached agreed to participate in the pilot study. 

### 3.2. Feedback on Questionnaire

The most frequent concerns raised by firefighters were the use of the abbreviation “PPE”. Firefighters were not familiar with this word and, often, needed an explanation. The other term was the use of “musculoskeletal”, which many were not familiar with either. The firefighters noted that the information required was successfully obtained via the questions, with no confusion or misinterpretation present. The station commanders and platoon commanders confirmed the appropriateness of the questionnaires and provided suggestions for terminology that all firefighters would understand, such as “physical injury”, and were able to provide examples of injuries and descriptions for certain questions.

### 3.3. Assessor and Instrument Reliability

The results from the pilot study indicated that all physical measures were reliably and consistently acquired (r = 0.912–0.998) (Table 1). 

Firefighters’ demographic characteristics, categorized by gender, are reported in Table 2. The mean age and years of experience of firefighters was 39.1 ± 9.6 years and 14.9 ± 10.5, respectively. The mean cardiorespiratory fitness for firefighters was over the recommended 42 mL·kg·min (12 METs). The overall completion time for the PAT was 418.2 ± 215.9 s. Regarding gender, the mean LBM for males was 61.9 ± 8.1 kg and 44.1 ± 3.5 kg for females (*p* < 0.001). The mean grip strength for males was 97.0 ± 24.1 kg and 61.4 ± 9.1 kg for females (*p* < 0.001). The mean leg strength for males was 118.5 ± 30.8 kg and 73.9 ± 11.4 kg for females (*p* < 0.001. The mean total completion time was 361.9 ± 169.8 s for males and 733.5 ± 178.9 s for females (*p* = 0.001), with males performing significantly better on all tasks. 

In Table 3 we report the differences between musculoskeletal injuries and weekly physical activity according to the PAT. The mean completion time for firefighters who had a history of musculoskeletal injures was 79.9 ± 65.3 s and for firefighters without a history of musculoskeletal injury, the completion time was 40.1 ± 27.7 s (*p* = 0.017) (Table 3).

In Table 4, we describe the prevalence of CVD risk factors, musculoskeletal injuries, suboptimal cardiorespiratory fitness and PAT results in our sample. According to the CVD risk factors, 19.4% of firefighters were aged, 26.1% were obese, 30.6% were hypertensive, 5.6% were diabetic, 30.6% were dyslipidaemic, 33.3% were cigarette smokers, 27.8% had a family history of heart disease and 66.7% were physically inactive. All CVD risk factors were categorized according to the ACSM guidelines [32]. A total of 61.1% of firefighters reported experiencing an injury throughout their carriers, with the most prevalent injury being the ankle and foot (21.7%). About half of firefighters (47.2%) did not meet the minimum recommended cardiorespiratory fitness requirement of 42 mL·kg·min for firefighting, while 69.7% of firefighters did not meet the minimum time requirement of 580 s for PAT. 

As seen in Table 5, there were significant negative correlations between LBM and total completion time (r = −0.725, *p* < 0.001), between VO_2max_ and total completion time (r = −0.593, *p* < 0.001), between grip strength and total completion time (r = −0.571, *p* < 0.001) and between leg strength and total completion time (r = −0.484, *p* = 0.004). In addition, there were significant positive correlations between BF% and the step-up (r = 0.503, *p* = 0.003), the charged hose drag and pull (r = 0.368, *p* = 0.035), and ladder raise and extension (r = 0.363, *p* = 0.038). Lean body mass, grip strength and leg strength were significantly and negatively correlated to all PAT tasks. In addition, VO_2max_ was significantly and negatively correlated to all occupational tasks, except the stair climb. Finally, there was a significant negative correlation between knee discomfort and the step-up (r = −0.350, *p* = 0.046), and between foot and ankle discomfort and the step-up (r = −0.364, *p* = 0.037).

## 4. Discussion

The objectives of the current study were to explore possible logistic or administrative obstacles the researchers would face in a similar larger study; determine the intra-assessor reliability of selected research equipment and survey instrument; determine the prevalence of CVD risk factors, musculoskeletal injuries, poor cardiorespiratory fitness and physical ability test (PAT) failures; and to explore the extent to which CVD risk factors and health metrics, musculoskeletal health and physical fitness are related to occupational performance in firefighters. The pilot study allowed us to determine effective methods of recruiting participants to participate in the larger study and plan and coordinate with fire department staff. The equipment was shown to be reliable and suitable for research purposes (r > 0.9). Face validation and content validation [39,40] of the questionnaire was successful, and accurately retrieved the data that was required from the firefighters. The pilot study indicated that the required information, such as demographics, CVD risk factors and health metrics, musculoskeletal health, physical fitness and PAT measures could be successfully obtained from the research equipment and instruments used. The pilot study indicated that the battery of tests would take approximately 40 to 55 min per firefighter, with three to four firefighters being tested simultaneously, which is feasible for the large study. Hypertension (30.6%), dyslipidaemia (33.3%), obesity (36.1%) and physical inactivity (66.7%) were the most prevalent CVD risk factors, with the prevalence of hypertension [16,21,41,42,43,44], dyslipidaemia [16,20,21,45] and physical inactivity [46,47,48] being higher than previous studies, and obesity prevalence similar to previous research [15,16,17,19,21]. The majority of firefighters (61.1%) reported having experienced musculoskeletal injuries, with the ankle and foot being the most frequently injured area (21.7%), which is consistent with previous literature [9]. In addition, 47.2% had not met the minimum recommended cardiorespiratory fitness level of 42 mL·kg·min based on estimated measures. Furthermore, 69.7% of firefighters did not meet the minimum time required to pass the PAT. Further, lean body mass, estimated VO2max, grip strength and leg strength were most related to individual tasks and total completion time for the PAT, which was consistent with previous literature [12,49].

### 4.1. Challenges Encountered, Feedback from Firefighters and Future Planning 

The firefighters indicated that the length of the questionnaire was a concern, with many of the questions being perceived as repetitive. Language used was important in the questionnaire, as several of the terms were confusing to the firefighters, and were changed to simpler terms based on suggestions provided. This feedback provided valuable information for the procedures for the full study, such as a much shorter questionnaire containing simpler terms and phrases. The researchers noted that firefighters required guidance on the self-administered questionnaire, with a designated researcher tasked to ensure that the questionnaire were answered correctly, and that all questions from the firefighters were answered succinctly. Potential obstacles were identified with the PAT, most attributed to researchers needing to ensure that time measuring was initiated at the onset of each activity, and that firefighters follow the correct order for the testing. For the full study, all these obstacles will be addressed to ensure efficiency and standardization of the testing. The validation of the research equipment and instruments was successful, as the data required were successfully obtained from the firefighters.

### 4.2. Preliminary Results 

The non-exercise estimation of VO2max formula that was used in the current study had been validated in a previous study, where it was shown to be reliable when compared to the Astrand submaximal bike test [36]. In addition, the results of that study indicated that the estimated VO2max was a moderately significant predictor of Astrand submaximal VO2max (r = 0.688, *p* < 0.001). Moreover, estimated VO2max has previously been used in studies conducted on firefighters [50,51]. A study investigated the accuracy of the non-exercise estimation of the VO2max method compared to the cooper 12 min test, and reported moderate to high reliability/specificity (71.5%) in firefighters [52]. Another study reported that moderate to moderate correlations existed between non-exercise estimation of VO2max and the Gerkin treadmill test (r = 0.69) and the Queen’s College step test (r = 0.51) [53]. The results in the current study indicated that 47.2% of firefighters did not meet the minimum requirement of 42 mL·kg·min for firefighting, as recommended by many researchers. A study by Houck et al. [54] reported that only 27.5% of full-time Urban and Wildland firefighters from New Mexico met the minimum recommended cardiorespiratory fitness level, substantially lower than the current pilot study. Comparably, Baur et al. [55] reported that 56.3% career firefighters in the United States did not meet the minimum recommendation of 42 mL·min·kg. In the current results, 69.7% of firefighters failed to complete the PAT in under 540 s, as stipulated in the firefighters’ health and wellness policy document. Stevenson et al. [56] conducted three firefighting tasks, namely, the ladder lift, the ladder lower, and the ladder extension, and found that 61% of firefighters passed the ladder lift, 77% passed the ladder lower and 71% passed the ladder extension. Von Heimburg et al. [57] found that 7.9% of firefighters failed to meet the requirements to pass the occupational simulation [57], considerably fewer than the present study, and was composed of the puzzle, hose dragging, hose connection and disconnection, heavy can carry, the heat chamber and the “retreat”. This may be due to the participants in the study having performed the occupational simulation protocol multiple times [57], as opposed to the current study, where firefighters only performed the simulation once. This is supported in studies by Schonfeld et al. [58] and Stevenson et al. [59] that found that subsequent performances of a simulation protocol resulted in better performances. Furthermore, the current results showed that cardiorespiratory fitness was significantly related to all PAT tasks, except the step-up, and was related to overall completion time. This may, somewhat, explain why numerous firefighters failed to meet the minimum time requirements for the PAT, as many did not meet the minimum fitness requirements. Previous studies supported this finding, as studies have shown that cardiorespiratory fitness may be the most significant factor in occupational performance in firefighters [60,61,62]. 

There was a significant difference between all PAT tasks and overall completion time between male and female firefighters. This result is consistent with previous research [63,64,65,66]. This should be an important consideration when conducting the larger study, as gender differences in performance may be a significant confounder in the completion times. A study by von Heimburg et al. [57] indicated that body mass and height were significantly related to occupational performance in firefighters. Since males are generally heavier and taller than females, this would partly explain the difference in performance. The study noted that shorter males with a lower body mass performed significantly worse on the occupational performance tasks [57]. In addition, many firefighting simulation tests use standardized equipment, requiring all firefighters to perform the same tasks using the same equipment, such as an 80 kg victim drag, and does not account for the relative size or weight of the firefighter performing the duties [60,62,67]. Invariably, lighter and smaller firefighters would exert more effort for the same task compared to their heavier and taller counterparts. On average, males had 36% greater skeletal muscle mass than females, and more specifically, 40% more skeletal muscle mass than females in the upper body and 33% more in the lower body [68]. This may be another important consideration regarding the differences in performance between male and female firefighters.

The results indicated that age, obesity, LBM, cardiorespiratory fitness, grip strength and leg strength were significantly related to occupational performance, which is similar to the results reported in previous studies [12,60,61,62,67].

### 4.3. Trends with Correlations

Numerous *p*-values bordered on significance, suggesting they would likely result in significant findings if a larger sample size were studied. 

### 4.4. Strengths and Limitations

A strength of the study was that this pilot would inform the feasibility of a larger study. This study indicated that the equipment used, and intra-assessor reliability was suitable for use in the larger scale study. The preliminary results indicated that significant relationships existed between the variables in this pilot study, suggesting significant results for the larger scale study. A weakness of this study was that only one fire station was used for the pilot. In addition, very few female firefighters partook in this study. The relatively small sample size of the firefighters in this study cannot be generalized to the larger firefighter population, motivating the implementation of the full-scale study. The selection of the participants was recruited via convenient sampling, rather than random sampling.

## 5. Conclusions

The current pilot study was successful in accomplishing the objectives that were set out, including the face and content validation of the survey questionnaire. The total time for completing the full battery of tests was established and deemed acceptable, especially as up to four firefighters can progress through the testing battery simultaneously. The assessors involved were given opportunity to familiarize themselves with the tests and the equipment and test–retest reliability showed high validity and reliability for use in the larger study. The preliminary results indicated that hypertension (30.6%) dyslipidaemia (33.3%), obesity (36.1%) and physical inactivity (66.7%) were the most prevalent CVD risk factors, with the majority of firefighters failing to meet the minimum required cardiorespiratory fitness level (47.2%) and required PAT completion time (69.7%). Cardiorespiratory fitness, lean body mass and grip and leg strength were significantly related to PAT tasks, and total completion time. We conclude that the planned larger study is feasible with no significant changes needed regarding the design and methodology. 

### Recommendations 

The questionnaire should be shortened and repetitive questions should be removed. The larger study should attempt to include more female firefighters to strengthen the generalizability of findings to females in the CoCTFRS. 

## Figures and Tables

**Table 1:** Assessor and instrument reliability and validity.

Variable	N	r
Body mass (kg)	36	0.988
Height (cm)	36	0.994
Bodyfat (%)	36	0.966
Lean body mass (kg)	36	0.975
Waist circumference (cm)	36	0.953
Hip circumference (cm)	36	0.967
Systolic blood pressure (mm Hg)	34	0.920
Diastolic blood pressure (mm Hg)	34	0.912
Total cholesterol (mmol·L^−1^)	10	0.997
Low-density lipoprotein (mmol·L^−1^)	10	0.999
High-density lipoprotein (mmol·L^−1^)	10	0.999
Triglycerides (mmol·L^−1^)	10	0.995
Non-fasting blood glucose (mmol·L^−1^)	10	0.998

Note: kg—kilogram; cm—centimetre; %—percentage; mm Hg—millimetres mercury; mmol·L^−1^—millimoles per litre; r—Pearson’s correlation coefficient.

**Table 2:** Demographic characteristics of firefighters according to gender.

Variable	Total Firefighters		Male		Female		*p*-Value
	N	$\bar{X}$ ± SD	N	$\bar{X}$ ± SD	N	$\bar{X}$ ± SD	
Age (years)	36	39.1 ± 9.6	30	38.6 ± 10.0	6	41.2 ± 7.6	0.312
Years of experience (years)	36	14.9 ± 10.5	30	15..3 ± 10.9	6	13.0 ± 8.6	0.717
Body mass (kg)	36	83.4 ± 14.4	30	85.9 ± 14.0	6	70.6 ± 8.3	0.010 *
Stature (cm)	36	173.9 ± 10.3	30	176.9 ± 8.2	6	159.3 ± 7.1	<0.001
BMI (kg·m^−2^)	36	27.6 ± 4.1	30	27.5 ± 4.1	6	27.9 ± 4.4	0.820
Waist circumference (cm)	36	94.0 ± 11.5	30	95.6 ± 11.2	6	86.0 ± 10.8	0.094
Hip circumference (cm)	36	106.4 ± 7.3	30	105.9 ± 7.2	6	109.2 ± 7.8	0.394
Bodyfat percentage (%)	36	24.9 ± 9.0	30	23.1 ± 8.3	6	34.2 ± 7.0	0.005 **
Lean body mass (kg)	36	58.9 ± 10.1	30	61.9 ± 8.1	6	44.1 ± 3.5	<0.001
Systolic blood pressure (mm Hg)	36	130.7 ± 11.9	30	129.7 ± 11.2	6	135.3 ± 15.2	0.418
Diastolic blood pressure (mm Hg)	36	80.1 ± 9.6	30	78.6 ± 8.8	6	87.2 ± 10.9	0.064
Total cholesterol (mmol·L^−1^)	36	4.6 ± 0.9	30	4.6 ± 0.9	6	4.7 ± 0.8	0.725
Low-density lipoprotein (mmol·L^−1^)	36	2.9 ± 0.8	30	2.7 ± 0.8	6	2.7 ± 0.6	0.664
High-density lipoprotein (mmol·L^−1^)	36	1.2 ± 0.3	30	1.2 ± 0.2	6	1.6 ± 0.3	0.741
Triglycerides (mmol·L^−1^)	36	1.6 ± 1.1	30	1.8 ± 1.1	6	1.0 ± 0.3	0.078
Non-fasting blood glucose (mmol·L^−1^)	36	5.2 ± 0.8	30	5.3 ± 0.8	6	4.7 ± 0.6	0.052
Heart rate variability (ms)	34	872.7 ± 165.1	28	877.8 ± 170.4	6	849.0 ± 149.4	0.644
Standard deviation of the N-N intervals (ms)	34	49.5 ± 27.9	28	51.1 ± 29.9	6	42.0 ± 16.7	0.741
Low frequency (Hz)	34	0.08 ± 0.03	28	0.09 ± 0.03	6	0.07 ± 0.02	0.145
High frequency (Hz)	34	0.21 ± 0.06	28	0.20 ± 0.05	6	0.25 ± 0.06	0.074
LF/HF ratio (ms)	34	4.14 ± 6.44	28	4.78 ± 6.95	6	1.2 ± 0.6	0.091
Physical fitness							
VO_2max_ (L·min)	34	3.6 ± 0.2	28	3.6 ± 0.3	6	3.5 ± 0.2	0.838
VO_2max_ (mL·kg·min)	34	44.0 ± 5.9	28	44.3 ± 6.2	6	43.3 ± 5.4	0.768
Grip strength (kg)	35	93.7 ± 21.0	29	97.0 ± 24.1	6	61.4 ± 9.1	<0.001 **
Leg strength (kg)	35	110.9 ± 33.0	29	118.5 ± 30.8	6	73.9 ± 11.4	<0.001 **
Push-up (RPM)	35	32.8 ± 13.9	29	33.2 ± 14.3	6	31.0 ± 12.9	0.815
Sit-up (RPM)	35	27.4 ± 9.8	29	28.6 ± 9.6	6	21.5 ± 9.2	0.093
Sit-and-reach (cm)	35	41.6 ± 8.6	29	41.1 ± 9.2	6	43.8 ± 4.4	0.535
Shoulder flexibility (R)	35	2.3 ± 0.9	29	2.2 ± 1.0	6	2.8 ± 0.4	0.272
Shoulder flexibility (L)	35	2.0 ± 1.0	29	1.9 ± 1.1	6	2.2 ± 0.8	0.815
Physical ability test							
Step-up (s)	33	72.1 ± 26.5	28	65.3 ± 11.3	5	110.0 ± 51.5	0.016 *
Charged hose drag and pull (s)	33	100.3 ± 55.5	28	82.6 ± 32.2	5	199.6 ± 55.9	<0.001 **
Ladder raise and extension (s)	33	78.8 ± 48.5	28	65.0 ± 29.5	5	156.2 ± 64.5	<0.001 **
Equipment carry (s)	33	60.0 ± 41.3	28	54.4 ± 41.1	5	91.8 ± 28.4	0.006 **
Forcible entry (s)	33	42.7 ± 37.2	28	38.2 ± 36.4	5	67.7 ± 34.6	0.039 *
Rescue drag (s)	33	66.3 ± 58.0	28	56.4 ± 52.2	5	135.3 ± 54.9	0.005 **
Total Time (s)	33	418.2 ± 215.9	28	349.4 ± 179.7	5	733.5 ± 178.9	0.001 **

Note: * indicates statistical significance < 0.05; ** indicates statistical significance < 0.01. $\bar{X}$—mean; SD—standard deviation; kg—kilogram; cm—centimetre; %—percentage; mm Hg—millimetres mercury; mmol·L^−1^—millimoles per litre; ms—milliseconds; Hz—Hertz; L·min—litres per minute; mL·kg·min– millilitres per minute per kilogram; RPM—repetitions per minute; s—seconds.

**Table 3:** Differences between physical ability test based on gender, injury history and weekly physical activity.

	Injuries			Weekly Physical Activity		
Physical Ability Test	Injured $\bar{X}$ ± SD	Never Injured $\bar{X}$ ± SD	*p*-Value	Vigorously Active $\bar{X}$ ± SD	Moderately Active $\bar{X}$ ± SD	*p*-Value
Step-up (s)	72.7 ± 20.3	71.1 ± 35.9	0.228	64.8 ± 14.7	76.3 ± 30.9	0.069
Charged hose drag and pull (s)	106.1 ± 53.2	90.1 ± 60.4	0.213	86.5 ± 45.6	108.2 ± 60.1	0.075
Ladder raise and extension (s)	82.9 ± 44.6	71.8 ± 56.2	0.131	67.6 ± 37.3	85.2 ± 53.7	0.258
Equipment carry (s)	68.7 ± 48.6	44.8 ± 16.7	0.104	61.3 ± 60.6	59.3 ± 26.6	0.104
Forcible entry (s)	48.1 ± 40.9	33.2 ± 28.5	0.518	40.4 ± 43.6	43.9 ± 34.1	0.326
Rescue drag (s)	79.9 ± 65.3	40.1 ± 27.7	0.017 *	61.9 ± 74.9	68.8 ± 47.2	0.136
Total Time (s)	458.4 ± 227.7	298.2 ± 203.4	0.069	382.6 ± 249.6	438.6 ± 197.8	0.082

Note: * indicates statistical significance < 0.05; $\bar{X}$—mean; SD—standard deviation; s—seconds; ±—standard deviation; *p*—significance level.

**Table 4:** Prevalence of CVD risk factors, musculoskeletal injuries, suboptimal physical fitness, and physical ability test failures in firefighters.

Variable	N	%
Aged (years)	7	19.4
Obesity (kg·m^−2^)	13	36.1
Central obesity (cm)	9	25.0
Hypertensive (mm Hg)	11	30.6
Diabetic (mmol·L^−1^)	2	5.6
Dyslipidaemia (mmol·L^−1^)	11	30.6
Cigarette smoking	12	33.3
Physical inactivity (min)	24	66.7
Family history	10	27.8
Musculoskeletal injuries	22	61.1
Neck	1	4.3
Shoulder	3	13.0
Elbow	1	4.3
Wrist and hand	1	4.3
Lower back	4	17.4
Knee	3	13.0
Ankle and foot	5	21.7
Multiple injuries	4	17.4
Suboptimal cardiorespiratory fitness (est. VO_2max_) #	17	47.2
Physical ability test time (s)	23	69.7

Note: kg—kilogram; cm—centimetre; %—percentage; mm Hg—millimetres mercury; mmol·L^−1^—millimoles per litre; ms—milliseconds; Hz—Hertz; L·min (estimated)—litres per minute; mL·kg·min (estimated)—millilitres per minute per kilogram (Est.); RPM—repetitions per minute; s—seconds. #—cardiorespiratory fitness defined as an estimated VO_2max_ of 42 mL·kg·min or 12 metabolic equivalents.

**Table 5:** Associations between CVD risk factors, heart rate variability, musculoskeletal health, physical fitness, and occupational performance (defined as time to complete each task and overall PAT completion time).

	1	2	3	4	5	6	7
CVD risk factors							
Age	0.302	0.373 *	0.084	0.208	0.145	0.056	0.241
Body mass index	0.391 *	0.067	0.081	−0.026	0.050	−0.166	0.058
Bodyfat percentage	0.503 **	−0.368 *	0.363	0.199	0.296	0.160	0.369 *
Lean body mass	−0.376 *	−0.690 **	−0.693 **	−0.642 **	−0.580 **	−0.697	−0.725 **
Systolic blood pressure	0.187	0.039	0.118	0.116	−0.064	0.021	0.104
Diastolic blood pressure	0.394 *	0.261	0.278	0.225	−0.002	0.174	0.272
Non-fasting blood glucose	−0.207	−0.069	−0.184	−0.008	−0.051	−0.040	−0.092
Total cholesterol	0.359 *	0.304	0.289	0.201	0.016	0.240	0.257
Heart rate variability							
Heart rate variability (N-N interval)	−0.142	−0.168	−0.048	−0.099	0.015	−0.278	−0.133
SDNN	−0.455 *	−0.329	−0.172	−0.166	0.071	−0.146	−0.225
RMSSD	−0.368 *	−0.219	−0.085	−0.109	0.155	−0.111	−0.126
Low frequency range	−0.264	−0.384 *	−0.377 *	−0.322	−0.302	−0.214	−0.365 *
High frequency range	0.243	0.438 *	0.338	0.386 *	0.466 **	0.273	0.398 *
Stress index	0.459 **	0.367 *	0.188	0.218	−0.018	0.165	0.261
Physical fitness							
Estimated VO_2max_ #	−0.258	−0.667 **	−0.459 **	−0.541 **	−0.395 *	−0.626 **	−0.593 **
Grip strength	−0.412 *	−0.589 **	−0.597 **	−0.527 **	−0.384 *	−0.554 **	−0.571 *
Leg strength	−0.301	−0.530 **	−0.466 **	−0.460 **	−0.400 *	−0.457 *	−0.484 **
Push-up	−0.201	−0.288	−0.085	−0.232	−0.215	−0.248	−0.222
Sit-ups	−0.354 *	−0.460 *	−0.189	−0.202	−0.289	−0.154	−0.308
Sit-and-reach	−0.206	−0.006	−0.041	−0.004	−0.105	−0.035	−0.059
Musculoskeletal health							
Neck	0.269	0.083	0.225	−0.115	0.015	−0.090	−0.067
Shoulder	0.107	−0.230	0.007	0.022	−0.306	−0.051	−0.083
Upper back	−0.003	0.082	0.144	0.009	0.149	0.123	0.115
Upper arm	0.230	0.029	0.018	−0.057	−0.024	0.088	0.084
Lower back	0.230	0.029	0.018	−0.057	−0.024	0.088	0.084
Forearm and wrist	−0.222	−0.201	−0.038	−0.127	−0.054	0.032	−0.080
Hip/buttocks	0.172	−0.019	0.002	−0.077	−0.174	−0.027	−0.033
Thigh	0.195	−0.195	0.056	−0.167	−0.139	0.029	−0.037
Knee	0.350 *	−0.097	−0.075	−0.136	0.003	0.195	−0.002
Lower leg	0.195	−0.195	0.056	−0.167	−0.139	0.029	−0.037
Foot and ankle	0.364 *	−0.064	0.101	0.094	0.093	0.140	0.113

Note: * indicates statistical significance < 0.05; ** indicates statistical significance <0.01; #—indicates VO_2max_ estimation from non-exercise equation. SDNN—standard deviation of normal-to-normal intervals; RMSSD—root mean square of successive differences between normal heartbeats; 1—step-up; 2—charged hose and pull; 3—ladder raise and extension; 4—equipment carry; 5—forcible entry; 6—rescue drag; 7—total completion time (overall PAT duration).

## Data Availability

The data presented in this study are available on request from the corresponding author. The data are not publicly available due to this being an ongoing study, and the POPIA act, which restricts the access to private information being made publicly available.

## References

[1] Smith D.L., Barr D.A., Kales S.N. (2013). Extreme Sacrifice: Sudden Cardiac Death in the US Fire Service. Extrem. Physiol. Med..

[2] Soteriades E.S., Smith D.L., Tsismenakis A.J., Baur D.M., Kales S.N. (2011). Cardiovascular Disease in US Firefighters: A Systematic Review. Cardiol. Rev..

[3] Smith D.L., DeBlois J.P., Kales S.N., Horn G.P. (2016). Cardiovascular Strain of Firefighting and the Risk of Sudden Cardiac Events. Exerc. Sport Sci. Rev..

[4] Marcel-Millet P., Ravier G., Grospretre S., Gimenez P., Freidig S., Groslambert A. (2018). Physiological Responses and Parasympathetic Reactivation in Rescue Interventions: The Effect of the Breathing Apparatus. Scand. J. Med. Sci. Sport..

[5] Smith D.L., Haller J.M., Korre M., Sampani K., Porto L.G.G., Fehling P.C., Christophi C.A., Kales S.N. (2019). The Relation of Emergency Duties to Cardiac Death Among US Firefighters. Am. J. Cardiol..

[6] Farioli A., Yang J., Teehan D., Baur D.M., Smith D.L., Kales S.N. (2014). Duty-Related Risk of Sudden Cardiac Death among Young US Firefighters. Occup. Med..

[7] Yang J., Teehan D., Farioli A., Baur D.M., Smith D., Kales S.N. (2013). Sudden Cardiac Death among Firefighters ≤ 45 Years of Age in the United States. Am. J. Cardiol..

[8] Phelps S.M., Drew-Nord D.C., Neitzel R.L., Wallhagen M.I., Bates M.N., Hong O.S. (2018). Characteristics and Predictors of Occupational Injury Among Career Firefighters. Workplace Health Saf..

[9] Frost D.M., Beach T.A.C., Crosby I., McGill S.M. (2015). Firefighter Injuries Are Not Just a Fireground Problem. Work.

[10] Smith D.L. (2011). Firefighter Fitness: Improving Performance and Preventing Injuries and Fatalities. Curr. Sport. Med. Rep..

[11] Yu C.C.W., Au C.T., Lee F.Y.F., So R.C.H., Wong J.P.S., Mak G.Y.K., Chien E.P., McManus A.M. (2015). Association Between Leisure Time Physical Activity, Cardiopulmonary Fitness, Cardiovascular Risk Factors, and Cardiovascular Workload at Work in Firefighters. Saf. Health Work.

[12] Chizewski A., Box A., Kesler R., Petruzzello S.J. (2021). Fitness Fights Fires: Exploring the Relationship between Physical Fitness and Firefighter Ability. Int. J. Environ. Res. Public Health.

[13] Mehrdad R., Movasatian F., Momenzadeh A.S. (1970). Fitness for Work Evaluation of Firefighters in Tehran. Acta Med. Iran..

[14] Donovan R., Nelson T., Peel J., Lipsey T., Voyles W., Israel R.G. (2009). Cardiorespiratory Fitness and the Metabolic Syndrome in Firefighters. Occup. Med..

[15] Ras J., Leach L. (2021). Prevalence of Coronary Artery Disease Risk Factors in Firefighters in the City of Cape Town Fire and Rescue Service–A Descriptive Study. J. Public Health Res..

[16] Savall A., Charles R., Bertholon A., Gramont B., Trombert B., Barthélémy J.C., Roche F. (2020). Volunteer and Career French Firefighters: Cardiovascular Risk Factors and Cardiovascular Risk Assessment. Eur. J. Prev. Cardiol..

[17] Martin Z.T., Schlaff R.A., Hemenway J.K., Coulter J.R., Knous J.L., Lowry J.E., Ode J.J. (2019). Cardiovascular Disease Risk Factors and Physical Fitness in Volunteer Firefighters. Int. J. Exerc. Sci..

[18] Mathias K.C., Bode E.D., Stewart D.F., Smith D.L. (2020). Changes in Firefighter Weight and Cardiovascular Disease Risk Factors over Five Years. Med. Sci. Sport. Exerc..

[19] Moffatt S.M., Stewart D.F., Jack K., Dudar M.D., Bode E.D., Mathias K.C., Smith D.L. (2021). Cardiometabolic Health among United States Firefighters by Age. Prev. Med. Rep..

[20] Bode E.D., Mathias K.C., Stewart D.F., Moffatt S.M., Jack K., Smith D.L. (2021). Cardiovascular Disease Risk Factors by BMI and Age in United States Firefighters. Obesity.

[21] Smith D.L., Graham E., Stewart D., Mathias K.C. (2020). Cardiovascular Disease Risk Factor Changes over 5 Years among Male and Female US Firefighters. J. Occup. Environ. Med..

[22] Ras J., Leach L. (2022). Firefighters’ Health Knowledge, Cardiovascular Disease Risk Factors and Sociodemographic Characteristics as Predictors of Firefighters Attitudes Toward Health. J. Occup. Environ. Med..

[23] Dobson M., Choi B., Schnall P.L., Wigger E., Garcia-Rivas J., Israel L., Baker D.B. (2013). Exploring Occupational and Health Behavioral Causes of Firefighter Obesity: A Qualitative Study. Am. J. Ind. Med..

[24] Bucala M., Sweet E. (2019). Obesity in the Fire Service: An inside Look at the Perceptions of Firefighters towards Obesity and Other Health Issues. Res. Sq..

[25] Ras J., Leach L. (2022). Relationship Between Physical Activity, Coronary Artery Disease Risk Factors and Musculoskeletal Injuries in the City of Cape Town Fire and Rescue Service. INQUIRY J. Health Care Organ. Provis. Financ..

[26] Geeta A., Jamaiyah H., Safiza M.N., Khor G.L., Kee C.C., Ahmad A.Z., Suzana S., Rahmah R., Faudzi A. (2009). Reliability, Technical Error of Measurements and Validity of Instruments for Nutritional Status Assessment of Adults in Malaysia. Singap. Med. J..

[27] Koo T.K., Li M.Y. (2016). A Guideline of Selecting and Reporting Intraclass Correlation Coefficients for Reliability Research. J. Chiropr. Med..

[28] Perini T.A., de Oliveira G.L., Ornelia J.S., de Oliveira F.P. (2005). Technical Error of Measurement in Anthropometry. Rev. Bras. Med. Esporte.

[29] Bohlmann I.M., Mackinnon S., Kruger H., Leach L., van Heerden J., Cook I., Noakes T., Lambert E. (2001). Is the International Physical Activity Questionnaire (IPAQ) Valid and Reliable in the South African Population?. Med. Sci. Sport. Exerc..

[30] Sanda B., Vistad I., Haakstad L.A.H., Berntsen S., Sagedal L.R., Lohne-Seiler H., Torstveit M.K. (2017). Reliability and Concurrent Validity of the International Physical Activity Questionnaire Short Form among Pregnant Women. BMC Sport. Sci. Med. Rehabil..

[31] Hedge A., Morimoto S., McCrobie D. (1999). Effects of keyboard tray geometry on upper body posture and comfort. Ergonomics.

[32] Liguori G., American College of Sports Medicine (2021). ACSM’s Guidelines for Exercise Testing and Prescription.

[33] Bastianelli K., Ledin S., Chen J. (2017). Comparing the Accuracy of 2 Point-of-Care Lipid Testing Devices. J. Pharm. Pract..

[34] Billman G.E., Huikuri H.V., Sacha J., Trimmel K. (2015). An Introduction to Heart Rate Variability: Methodological Considerations and Clinical Applications. Front. Physiol..

[35] Tomes C., Schram B., Orr R. (2020). Relationships Between Heart Rate Variability, Occupational Performance, and Fitness for Tactical Personnel: A Systematic Review. Front. Public Health.

[36] Rexhepi A.M., Brestovci B. (2014). Prediction of VO2max Based on Age, Body Mass, and Resting Heart Rate. Hum. Mov..

[37] Bianco A., Lupo C., Alesi M., Spina S., Raccuglia M., Thomas E., Paoli A., Palma A. (2015). The Sit up Test to Exhaustion as a Test for Muscular Endurance Evaluation. Springerplus.

[38] Anderson M.K., Hall S.J., Martin M. (2004). Foundations of Athletic Training: Prevention, Assessment, and Management.

[39] Mosier C.I. (1947). A Critical Examination of the Concepts of Face Validity. Educ. Psychol. Meas..

[40] Sireci S.G. (1998). The Construct of Content Validity. Soc. Indic. Res..

[41] Choi B.K., Schnall P., Dobson M. (2016). Twenty-Four-Hour Work Shifts, Increased Job Demands, and Elevated Blood Pressure in Professional Firefighters. Int. Arch. Occup. Environ. Health.

[42] Noh J., Lee C.J., Hyun D.S., Kim W., Kim M.J., Park K.S., Koh S., Chang S.J., Kim C., Park S. (2020). Blood Pressure and the Risk of Major Adverse Cardiovascular Events among Firefighters. J. Hypertens..

[43] Soteriades E.S., Hauser R., Kawachi I., Christiani D.C., Kales S.N. (2008). Obesity and Risk of Job Disability in Male Firefighters. Occup. Med..

[44] Khaja S.U., Mathias K.C., Bode E.D., Stewart D.F., Jack K., Moffatt S.M., Smith D.L. (2021). Hypertension in the United States Fire Service. Int. J. Environ. Res. Public Health.

[45] Smith D.L., Fehling P.C., Frisch A., Haller J.M., Winke M., Dailey M.W. (2012). The Prevalence of Cardiovascular Disease Risk Factors and Obesity in Firefighters. J. Obes..

[46] Durand G., Tsismenakis A.J., Jahnke S.A., Baur D.M., Christophi C.A., Kales S.N. (2011). Firefighters’ Physical Activity: Relation to Fitness and Cardiovascular Disease Risk. Med. Sci. Sport. Exerc..

[47] Baur D.M., Christophi C.A., Cook E.F., Kales S.N. (2012). Age-Related Decline in Cardiorespiratory Fitness among Career Firefighters: Modification by Physical Activity and Adiposity. J. Obes..

[48] Gendron P., Lajoie C., Laurencelle L., Trudeau F. (2018). Cardiovascular Disease Risk Factors in Québec Male Firefighters. J. Occup. Environ. Med..

[49] Rhea M.R., Alvar B.A., Gray R. (2004). Physical Fitness and Job Performance of Firefighters. J. Strength Cond. Res..

[50] Porto L.G.G., Schmidt A.C.B., de Souza J.M., Nogueira R.M., Fontana K.E., Molina G.E., Korre M., Smith D.L., Junqueira L.F., Kales S.N. (2019). Firefighters’ Basal Cardiac Autonomic Function and Its Associations with Cardiorespiratory Fitness. Work.

[51] Poston W.S.C., Development N., Haddock C.K., Development N., Jahnke S.A., Development N., Jitnarin N., Development N., Development N., Tuley B.C. (2018). The Prevalence of Overweight, Obesity, and Substandard Fitness in a Population-Based Firefighter Cohort. J. Occup. Environ. Med..

[52] Segedi L.C., Saint-Martin D.R.F., da Cruz C.J.G., von Koenig Soares E.M.K., do Nascimento N.L., da Silva L.L., Nogueira R.M., Korre M., Smith D.L., Kales S. (2020). Cardiorespiratory Fitness Assessment among Firefighters: Is the Non-Exercise Estimate Accurate?. Work.

[53] Heydari P., Varmazyar S., Safari Variani A., Hashemi F., Ataei S.S. (2017). Correlation of Gerkin, Queen’s College, George, and Jackson Methods in Estimating Maximal Oxygen Consumption. Electron. Physician.

[54] Houck J.M., Mermier C.M., Beltz N.M., Johnson K.E., VanDusseldorp T.A., Escobar K.A., Gibson A.L. (2020). Physical Fitness Evaluation of Career Urban and Wildland Firefighters. J. Occup. Environ. Med..

[55] Baur D.M., Christophi C.A., Tsismenakis A.J., Cook E.F., Kales S.N. (2011). Cardiorespiratory Fitness Predicts Cardiovascular Risk Profiles in Career Firefighters. J. Occup. Environ. Med..

[56] Stevenson R.D.M., Siddall A.G., Turner P.F.J., Bilzon J.L.J. (2017). Physical Employment Standards for UK Firefighters. J. Occup. Environ. Med..

[57] von Heimburg E., Medbø J.I., Sandsund M., Reinertsen R.E. (2013). Performance on a Work-Simulating Firefighter Test versus Approved Laboratory Tests for Firefighters and Applicants. Int. J. Occup. Saf. Ergon..

[58] Schonfeld B.R., Doerr D.F., Convertino V.A. (1990). An Occupational Performance Test Validation Program for Fire Fighters at the Kennedy Space Center. J. Occup. Environ. Med..

[59] Stevenson R.D.M., Siddall A.G., Turner P.J.F., Bilzon J.L.J. (2019). Validity and Reliability of Firefighting Simulation Test Performance. J. Occup. Environ. Med..

[60] Skinner T.L., Kelly V.G., Boytar A.N., Peeters G., Rynne S.B. (2020). Aviation Rescue Firefighters Physical Fitness and Predictors of Task Performance. J. Sci. Med. Sport.

[61] Siddall A.G., Stevenson R.D.M., Turner P.J.F., Bilzon J.L.J. (2018). Physical and Physiological Performance Determinants of a Firefighting Simulation Test. J. Occup. Environ. Med..

[62] Michaelides M.A., Parpa K.M., Thompson J., Brown B. (2008). Predicting Performance on a Firefighter’s Ability Test from Fitness Parameters. Res. Q. Exerc. Sport.

[63] Misner J.E., Plowman S.A., Boileau R.A. (1987). Performance Differences between Males and Females on Simulated Firefighting Tasks. J. Occup. Med..

[64] Henderson N.D., Berry M.W. (2007). Field Measures of Strength and Fitness Predict Firefighter Performance on Physically Demanding Tasks. Pers. Psychol..

[65] Nazari G., MacDermid J.C., Sinden K.E., Overend T.J. (2018). The Relationship between Physical Fitness and Simulated Firefighting Task Performance. Rehabil. Res. Pract..

[66] Myhre L.G., Tucker D.M., Bauer D.H., Fisher Jr J.R., Grimm W.H. (1997). Relationship between Selected Measures of Physical Fitness and Performance of a Simulated Fire Fighting Emergency Task.

[67] Michaelides M.A., Parpa K.M., Henry L.J., Thompson G.B., Brown B.S. (2011). Assessment of Physical Fitness Aspects and Their Relationship to Firefighters’ Job Abilities. J. Strength Cond. Res..

[68] Janssen I., Heymsfield S.B., Wang Z., Ross R. (2000). Skeletal Muscle Mass and Distribution in 468 Men and Women Aged 18–88 Yr. J. Appl. Physiol..

